# Computational methods and tools to predict cytochrome P450 metabolism for drug discovery

**DOI:** 10.1111/cbdd.13445

**Published:** 2019-01-15

**Authors:** Jonathan D. Tyzack, Johannes Kirchmair

**Affiliations:** ^1^ EMBL‐EBI, Wellcome Genome Campus Cambridge UK; ^2^ Department of Chemistry University of Bergen Bergen Norway; ^3^ Computational Biology Unit (CBU) University of Bergen Bergen Norway; ^4^ Center for Bioinformatics Universität Hamburg Hamburg Germany

**Keywords:** cytochrome P450, drug discovery, enzyme–ligand interaction, machine learning, metabolism, metabolite structures, prediction, reactivity, sites of metabolism

## Abstract

In this review, we present important, recent developments in the computational prediction of cytochrome P450 (CYP) metabolism in the context of drug discovery. We discuss in silico models for the various aspects of CYP metabolism prediction, including CYP substrate and inhibitor predictors, site of metabolism predictors (i.e., metabolically labile sites within potential substrates) and metabolite structure predictors. We summarize the different approaches taken by these models, such as rule‐based methods, machine learning, data mining, quantum chemical methods, molecular interaction fields, and docking. We highlight the scope and limitations of each method and discuss future implications for the field of metabolism prediction in drug discovery.

## 1 INTRODUCTION

Understanding the metabolism of small molecules is of paramount importance to the drug discovery industry and mitigates the risk of costly late failure in drug development projects due to adverse ADMET properties. Modern experimental approaches enable the elucidation of ADMET properties at an unprecedented level of detail but remain costly and time‐consuming, so it is desirable to have efficient and reliable in silico methods in place (Kirchmair et al., 2015; Wilson, 2014). The most effective computational approaches allow the profiling of large datasets and enable the interactive optimization of lead compounds but at vastly lower expense. In the context of metabolism prediction, in silico tools are most commonly used for predicting substrates and inhibitors of metabolic enzymes, sites of metabolism (SoMs that is, metabolically labile atom positions in the substrate at which biotransformations are initiated) and structures of likely metabolites. These predictions can then be used as part of the multi‐parameter optimization drug discovery process, helping to satisfy stability constraints, increase in vivo half‐life and avoid toxic metabolites.

**Figure 1:**
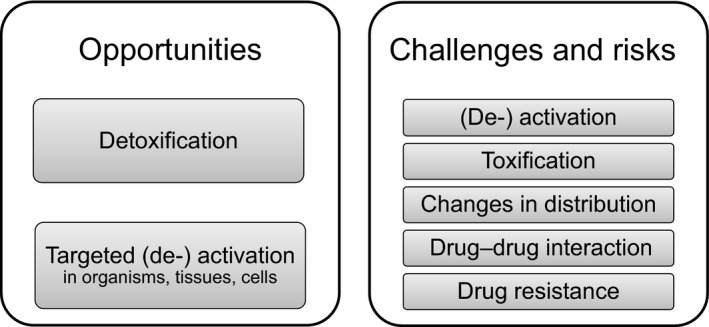
Opportunities, challenges and risks related to drug metabolism

Metabolic enzymes and systems have evolved to provide defense against xenobiotics (foreign and potentially hazardous molecules in our environment such as toxins and poisons) by transforming them into more readily excretable metabolites (Testa, 2014; Tyzack, Furnham, Sillitoe, Orengo, & Thornton, 2017). Pharmaceuticals and other molecules encountered through the course of modern life fall under the remit of these metabolic processes which operate on them in two broad categories: phase I metabolism involves making the molecule more polar and hydrophilic; and phase II involves conjugation with endogenous hydrophilic compounds. The net result is that metabolism is responsible for the clearance of about 75% of all drugs (Di, 2014) producing metabolites with different physicochemical, physiological, pharmacological, and toxicological properties (Kirchmair, et al., 2015; Kirchmair, Howlett, et al., 2013; Tyzack & Glen, 2014). Metabolism poses many challenges, but a more complete understanding also generates opportunities (Testa, Pedretti, & Vistoli, 2012), as summarized in Figure 1.

The most important enzymes in phase I belong to the cytochrome P450s (CYPs) since they produce the most first generation metabolites and have a high proportion of toxic/reactive metabolites (Testa et al., 2012). They are a family of heme‐containing enzymes ubiquitously found in animals, plants, fungi, and bacteria where at least 57 CYP isoforms have been documented in humans. The different CYP isoforms exhibit varying pocket sizes, shapes, binding surfaces, and flexibility, giving them different substrate specificity profiles and directing metabolism toward different parts of small molecules (Leach & Kidley, 2014; Mustafa, Yu, & Wade, 2014; Testa, 2014). Some CYP isoforms have remarkable ligand promiscuity driven in part by the size and plasticity of their binding sites where significant flexibility and conformational change has been revealed with molecular dynamics simulations (Mustafa et al., 2014). CYPs can be classified into two major classes: those involved in xenobiotic detoxification found mainly in the liver (such as the CYP2 and CYP3 families); and those involved in the biosynthesis of endogenous compounds such as sterols, fatty acids, eicosanoids, and vitamins (Guengerich, Waterman, & Egli, 2016; Rendic & Guengerich, 2015).

There are many factors that make understanding the action of CYP enzymes in vivo challenging, including expression patterns, inhibition levels, and genetic polymorphisms. Expression patterns vary significantly across organs, the highest human concentrations being found in the liver and small intestine, but expression is also influenced by gender, age, disease, stress, lifestyle, diet, and medication (Testa, 2014). These factors will all influence in vivo CYP expression and the rate of drug clearance. Furthermore, CYP inhibition and induction can be hugely influential, such as the flavonoid CYP inhibitors found in grapefruit juice that can result in higher drug concentrations than anticipated in the dosing regimen. Conversely, CYP induction can cause drug concentrations to fall below therapeutic levels, such as the dietary supplement St John's Wort, a potent inducer of CYP3A4 (Roby, Anderson, Kantor, Dryer, & Burstein, 2000). CYP genetic polymorphisms manifesting as loss or gain of function variants also need to be considered in the drug development process so that undue reliance is not placed on CYP isoforms that have known deficiencies in certain ethnic populations. These factors all contribute to an overall complex picture and make prediction of drug metabolism highly challenging but essential for drug discovery and development.

This review is intended to describe important contributions in the field of CYP metabolism prediction and cover more recent developments in this field but with a focus on methods that are freely available (Table 1). More comprehensive and complete lists of publications, software, and databases can be found elsewhere (Bezhentsev et al., 2016; Kar & Leszczynski, 2017; Kirchmair et al., 2015) with other reviews covering topics such as molecular dynamics, and QM modeling (Kirchmair et al., 2012; Shaik, Chen, Usharani, & Thiel, 2014; Williamson, 2014) and pharmacogenetics, pharmacoepigenetics, and clinical significance (Manikandan & Nagini, 2018) in more detail.

**Table 1:** Free in silico models for the prediction drug metabolism

Tool name	Description	Available as a free		
		Software package	Web service	At URL
Prediction of CYP specificity				
OpenVirtualToxLab (Vedani et al., 2015)	Combination of flexible docking with multi‐dimensional QSAR Predicts inhibitors and non‐inhibitors of CYP 1A2, 2A13, 2C9, 2D6, and 3A4	x		http://www.biograf.ch/data/projects/OpenVirtualToxLab.php
SwissADME (Daina et al., 2017)	Support vector machines Predicts inhibitors and non‐inhibitors of CYP 1A2, 2C9, 2C19, 2D6, and 3A4		x	http://www.swissadme.ch
CypRules (Shao et al., 2015)	Learning base model Predicts inhibitors and non‐inhibitors of CYP 1A2, 2C9, 2C19, 2D6, and 3A4		x	https://cyprules.cmdm.tw
CypReact (Tian et al., 2018)	Predicts substrates and non‐substrates of CYP 1A2, 2A6, 2B6, 2C8, 2C9, 2C19, 2D6, 2E1, and 3A4	x		https://bitbucket.org/Leon_Ti/cypreact
Prediction of sites of metabolism				
SMARTCyp (Rydberg, Gloriam, & Olsen, 2010; Rydberg, Gloriam, Zaretzki, et al., 2010)	Predicts SoMs for CYPs based on reaction energies derived from density functional theory	x	x	https://smartcyp.sund.ku.dk
SOMP (Rudik et al., 2015)	Predicts SoMs for five major CYPs and for UDP‐glucuronosyltransferases based on a Bayesian approach		x	http://www.way2drug.com/SOMP/
FAME 2 (Šícho et al., 2017)	Predicts SoMs for CYPs with extremely randomized trees	x		https://www.zbh.uni-hamburg.de/forschung/acm/software_datasets.html
XenoSite (Matlock et al., 2015; Zaretzki et al., 2013)	Predicts SoMs for CYPs with neural networks		x	http://swami.wustl.edu/xenosite
Prediction of metabolite structures				
SyGMa (Ridder & Wagener, 2008)	Expert‐curated rule set with empirical scoring of metabolites generated by phase I and phase II reactions	x^a^		https://github.com/3D-e-Chem/sygma
ToxTree (Patlewicz et al., 2008)	Set of biotransformation rules for CYPs applied to SoMs predicted with SMARTCyp	x		http://toxtree.sourceforge.net
MetaTox (Rudik et al., 2017)	Bayesian approach for five major CYPs and for UDP‐glucuronosyltransferases		x	http://www.way2drug.com/mg/

a Available also as a KNIME node.

## 2 METHODS FOR CYP SPECIFICITY PREDICTION

Understanding the specificity of individual CYP isoforms to bind small molecules can assist in the prediction of metabolic stability, enzyme inhibition, and drug–drug interactions. Docking methods explicitly model the substrate binding event, such as OpenVirtualToxLab (Vedani, Dobler, Hu, & Smieško, 2015) that uses flexible docking in combination with multi‐dimensional QSAR to predict the inhibition of five of the main xenobiotic metabolizing CYPs. However, these methods are computationally expensive, presenting challenges when attempting to incorporate them into software tools. For this reason, machine learning methods such as neural networks (Dai, Xu, Xiong, Liu, & Wei, 2016), support vector machines (SVMs) (Daina, Michielin, & Zoete, 2017) and random forests (Hunt, Segall, & Tyzack, 2018) have evolved as the mainstay for predicting enzyme specificity as they allow the modeling of the complex nonlinear relationships observed in large collections of enzyme–substrate interaction data. They differ in terms of the data on which they are trained, the descriptors used to represent the data, the scope of predictions, and the type of machine learning methodology employed. Machine learning models produce accurate results quickly and so lend themselves for usage in web applications where results can be returned in real time. However, the quantity and quality of the available data is often a limiting factor and determines the coverage and performance of these models (Kirchmair et al., 2015). So far they have not allowed the generation of sufficiently accurate regression models but only classification models (Gleeson et al., 2007).

When applying models for CYP specificity prediction, care must be taken to ensure that the predictive models are based on predicting enzyme–substrate interactions or enzyme–inhibitor interactions. SwissADME (Daina et al., 2017) is a web service that offers, among many other tools, SVM models for the prediction of inhibitors for the five major CYP isoforms (i.e., CYPs 1A2, 2C19, 2C9, 2D6, and 3A4). The classifiers were trained on data from the PubChem Bioassay 1851 dataset (Veith et al., 2009) using 50 molecular and physicochemical descriptors. CypRules (Shao et al., 2015) is another web service that predicts inhibitors and non‐inhibitors of the same major CYPs based on the same data. It utilizes decision trees in combination with the concept of information entropy.

In contrast, a downloadable java program called CypReact (Tian, Djoumbou‐Feunang, Greiner, & Wishart, 2018) classifies molecules as substrate or non‐substrate (but not inhibitor and non‐inhibitor) for nine major xenobiotic metabolizing isoforms. It uses a machine learning method called LBM (learning base model) based on many physicochemical and structural properties but applies feature selection to avoid over‐fitting. The training data of approximately one thousand compounds was compiled from several different sources.

There are also a number of commercial offerings for the prediction of enzyme inhibition and specificity. The StarDrop platform contains functionality called WhichP450 (Hunt et al., 2018) that identifies enzyme–substrate interactions for seven major xenobiotic metabolizing isoforms. It uses random forest models based on datasets manually extracted from the primary literature, where care was taken to annotate the training data with major and minor metabolizing isoforms for completeness. ADMET Predictor (Simulations Plus) has functionality to both predict inhibitors for five major drug metabolizing CYPs and substrates for nine CYP isoforms using data acquired from the BIOVIA Metabolite database, (BIOVIA Metabolite, n.d.) the Drugbank database (Wishart et al., 2018), and other public resources.

## 3 METHODS FOR SITE OF METABOLISM PREDICTION

There are two components to model in order to predict SoMs: the reactivity and accessibility of atoms in a molecule. Modern methods for SoM prediction follow different concepts to model these two components falling into two categories: structure‐based and ligand‐based. Structure‐based methods incorporate knowledge about the CYP enzyme and suffer from being computationally expensive. Consequently, ligand‐based methods trained on datasets of known (non‐) binders and metabolic sites are far more commonly applied in prediction tools. Ligand‐based methods can be further subdivided into the sub‐categories data mining, expert knowledge, and machine learning. The models presented in the following sections address reactivity and accessibility using various combinations of structure‐ and ligand‐based approaches.

### 3.1 Reactivity

The reactivity of different molecular fragments toward CYP‐mediated oxidation is the major determinant of SoMs. Reactivity can only really be classified as structural in terms of QM‐MM simulations that model the reaction in silico but the extremely high computational cost limits this approach to very detailed studies of a particular pathway with a particular substrate (Shaik et al., 2014). Consequently, reactivity is nearly always ligand‐based in the SoM prediction tools that will be described. Data mining, expert knowledge, and machine learning methods are almost exclusively ligand‐based, involving searching annotated ligand datasets, applying knowledge of ligand metabolism, or learning from ligand datasets respectively.

#### 3.1.1 Reactivity: Simulation‐based

QM methods allow the accurate investigation of reactivity but at substantial computational expense and it is common for investigators to carry out QM calculations on a predefined library of molecular fragments from which to match to query molecules. This technique was adopted as one component of SMARTCyp (Rydberg, Gloriam, & Olsen, 2010; Rydberg, Gloriam, Zaretzki, Breneman, & Olsen, 2010), which predicts SoMs based on activation energies calculated using density functional theory (DFT) calculations applied to a library of molecular fragments. The P450 Module within the commercial software stardrop adopts a different approach (Tyzack, Hunt, & Segall, 2016), directly modeling the reaction pathway using parameterized semi‐empirical AM1 QM methods. This enables activation energy estimates to be generated for each molecular fragment in the context of the molecular environment in which it resides, rather than treating each fragment as identical regardless of its neighboring atomic moieties. The semi‐empirical QM methods use a methoxy radical to simplify the calculations since modeling the heme group and its protein environment is more akin to full QM‐MM methods which are extremely computationally expensive and cannot be run routinely for SoM identification (Olsen, Rydberg, Rod, & Ryde, 2006; Shaik et al., 2014).

#### 3.1.2 Reactivity: Descriptor‐based

Other methods include reactivity descriptors as a component to approximate the hydrogen and electron abstraction processes fundamental to the CYP catalytic cycle but often require QM minimization to be carried out on a query molecule which carries significant computational expense. A number of studies have identified reactivity descriptors based on the energies of molecular orbitals (Mukherjee, Lal Gupta, & Jayaram, 2015; Tyzack, Williamson, Torella, & Glen, 2013) and hardness (Pragyan, Kesharwani, Nandekar, Rathod, & Sangamwar, 2014) as important metrics to help determine SoMs. The commercial software ADMET Predictor (Simulations Plus) uses reactivity descriptors based on Huckel charges and semi‐empirical molecular orbital calculations among others, and MetScore (Göller, Finkelmann, Goldmann, & Schneider, 2018) is based on quantum chemical partial charges.

### 3.2 Accessibility

Accessibility can be modeled either as structure‐based, using (pseudo‐) docking approaches to insert the molecule into the CYP binding pocket, or ligand‐based, using molecular fingerprints to approximate steric hindrance and orientational concerns. These approaches will be discussed in more detail alongside the reactivity metric they supplement.

#### 3.2.1 Accessibility: Structure‐based

Structure‐based methods explicitly model the accessibility criteria by placing the molecule into the CYP binding pocket. The output from these methods is easy to interrogate as they generate binding poses that can be inspected by the user and are rooted in the physical reality of explicitly modeling the binding event. Today, crystal structures are available for almost all CYP isoforms relevant to xenobiotic metabolism (Guengerich, 2014; Oostenbrink, 2014). Malleability of these enzymes, their complex interplay with water, and the hydrophobic character of their—in part—very large binding sites pose significant challenges to the application of structure‐based approaches, in particular docking. For this reason, structure‐based methods are primarily applied for the detailed investigation of the interaction of CYPs with individual compounds rather than for the profiling of small‐molecule libraries. In this setup, structure‐based methods can be particularly valuable for rationalizing distinct biological properties of enantiomers.

A large number of studies employing docking algorithms to determine the SoMs of small molecules have been published. One example from 2015 used Autodock Vina (Trott & Olson, 2010) to insert substrates into an ensemble of CYP 2C9 structures (Kingsley, Wilson, Essex, & Lill, 2015) and gave overall predictions by coupling with reactivities from SMARTCyp. The authors highlighted the importance of considering flexibility and sampling conformational space by the improvement in going from docking into a single structure to an ensemble of structures. Constrained docking has also been explored (Tyzack et al., 2013), reducing the size of the search space by fixing each ligand atom in turn in a position relevant to catalysis relative to the CYP heme and explicitly obtaining a score for each potential SoM. However, it is far more common for unconstrained docking to be applied, predicting SoMs as those that are in close proximity to the heme in the best pose(s), but placing reliance on the docking algorithm to fully explore conformational space rather than forcing it to consider each potential SoM as in the constrained workflow.

Another publication used the top three poses from in‐house docking software combined with reactivity descriptors based on molecular orbitals to build a classifier with good performance (Mukherjee et al., 2015). The docking method carries considerable computational expense since its estimation of binding free energies requires the calculation of partial charges from QM software and energy minimization with MD software, but a good correlation to experimental binding free energies was observed. The docking software GLIDE has also been combined with reactivity descriptors based on hardness to identify metabolically labile sites (Pragyan et al., 2014).

There are also several commercial packages available that make SoM predictions using CYP structural information. MetaSite (Cruciani et al., 2005) was one of the pioneers of structure‐based methods for SoM prediction and was developed into a commercial software package. It uses a pseudo‐docking approach where steric and chemical properties of CYPs such as hydrogen bonding or hydrophobic regions are described by molecular interaction fields (MIFs), calculated by placing various chemical probes in a grid system embedded over the CYP structure. Potential SoMs can be identified in a target molecule by aligning to the MIFs, coupled with matching molecular fragments to pre‐computed reactivity scores from QM approaches. IDSite (Li, Schneebeli, Bylund, Farid, & Friesner, 2011) samples the conformational space of CYPs as part of a flexible docking procedure with Glide (Friesner et al., 2004).

#### 3.2.2 Accessibility: Ligand‐based

Ligand‐based approaches have the benefit of speed and have been shown to perform at least as well as structural methods, although sometimes with less interpretability. They provide indirect information about the active site of a protein on the basis of the location of documented SoMs in the molecular graph, a concept rather like describing a glove by the properties of hands that will fit into it. There are various ways to encode the accessibility of molecular fragments using a 2D molecular graph such as the simple SPAN predictor used in SMARTCyp (Rydberg, Gloriam, Zaretzki, et al., 2010) which measures the relative distance to the “edge” of a molecule and was used in conjunction with pre‐computed reactivity scores. A different concept is the use of 3D alignments to derive the SoMs for a compound of interest from reference compounds with annotated SoMs (de Bruyn Kops, Friedrich, & Kirchmair, 2017). Typically, methods evaluating the accessibility of atom positions are combined with reactivity metrics to produce predictive models such as the P450 Module within StarDrop which contains orientational and steric descriptors derived from manually curated datasets from the primary literature.

### 3.3 Data mining

These methods encode the SoMs stored in metabolic databases in a manner that allows easy interrogation with molecular fragments in query molecules. One example is MetaPrint2D (Adams, 2010) that derives likelihoods of metabolic transformations for atoms in defined atomic environments by mining the BIOVIA Metabolite database, but is unfortunately no longer maintained. PROXIMAL (Yousofshahi, Manteiga, Wu, Lee, & Hassoun, 2015) uses a similar data mining of atomic sites approach but using the KEGG and DrugBank databases, where matches are scored by activity and abundance data obtained from the literature.

### 3.4 Machine learning

Machine learning software tools are popular since they can be routinely applied to large datasets early in the drug discovery process (Xiong et al., 2018). However, the size and quality of available data and the features used to describe them are of paramount importance in the development of these approaches. Much data are held in‐house within big pharmaceutical companies but there is a dearth of high‐quality SoM data in the public domain where the Zaretzki dataset (Zaretzki, Matlock, & Swamidass, 2013) (recently revised by de Bruyn Kops et al., 2017) remains the most comprehensive. Also, many of the datasets only record the major sites and metabolites and often significant secondary or tertiary data points are overlooked, leading to pessimistic false positive rates being reported when a non‐recorded SoM is predicted. Recent efforts at Optibrium to collate enlarged, high‐quality datasets from the literature highlighted this issue where it was observed that papers from different groups studying the same metabolic process varied in the completeness of the metabolic sites recorded although this data remain in‐house and forms the basis of the P450 module within StarDrop.

There can be a tendency with machine learning approaches to keep adding extra descriptors but this can lead to over‐fitting problems and hinder interpretability of the models. However, one study showed that 2D topological descriptors based on MOLPRINT2D fingerprints (Bender, Mussa, Glen, & Reiling, 2004) coupled with machine learning methods were sufficient to produce strong classification performance (Tyzack, Mussa, Williamson, Kirchmair, & Glen, 2014). Descriptors based on 2D atomic neighborhoods also form the basis of SOMP (Rudik, Dmitriev, Lagunin, Filimonov, & Poroikov, 2014) (available as a web server; Rudik, Dmitriev, Lagunin, Filimonov, & Poroikov, 2015) which uses Bayesian classification models trained on data from the BIOVIA Metabolite database and confirms that successful prediction models can be built without having to resort to a plethora of QM based descriptors.

FAME 2 (Šícho, de Bruyn Kops, Stork, Svozil, & Kirchmair, 2017) is an accurate and robust approach to the prediction of SoMs related to CYP metabolism. It is built on the strong foundations laid by its predecessor, FAME (Kirchmair, Williamson, et al., 2013), but utilizes an extremely randomized tree algorithm (rather than random forest) and a new type of circular fingerprint. At the same time, FAME 2 maintains the overall design ethos of stripping away complexity where it is not justified in terms of performance. The more descriptive topological descriptors of FAME 2 produce models that generalize well, making them applicable to molecular structures distant from those represented by the training data, such as complex natural products. The model was shown to obtain competitive performance with computationally more expensive methods based on density functional theory (Finkelmann, Göller, & Schneider, 2017; Šícho et al., 2017).

Another example of a free SoM predictor is Xenosite (Zaretzki et al., 2013), which was built on RS‐Predictor. Xenosite utilizes neural networks trained on topological, molecular, and quantum chemical descriptors coupled with SMARTCyp reactivities. In contrast to its predecessor, outputs are represented as probabilities rather than rank orderings. Xenosite is available as a web server (Matlock, Hughes, & Swamidass, 2015) and was extended to non‐CYP applications such as UGT‐mediated metabolism prediction (Dang, Hughes, Krishnamurthy, & Swamidass, 2016).

## 4 METHODS FOR METABOLITE STRUCTURE PREDICTION

Methods for metabolite structure prediction are dominated by knowledge‐based approaches. These approaches rely on cumulative knowledge acquired by drug discovery scientists over the years through observation and experimental work. Underlying these methods are a series of expert‐curated rules that represent SoMs and metabolic transformations, but when applied iteratively they can lead to an unmanageable combinatorial explosion of predictions. SyGMa (Ridder & Wagener, 2008) combines an expert‐curated rule set with empirical scoring to predict and rank the likely products of phase I and phase II metabolism. It was one of the earliest freely available offerings to generate metabolite structures but remains a highly effective and useable tool. More recently, in addition to being made available as a software package, SyGMa has been released as a KNIME (Berthold et al., 2018) node as part of the 3D‐e‐Chem project (Kooistra et al., 2018). ToxTree (Patlewicz, Jeliazkova, Safford, Worth, & Aleksiev, 2008) includes a module for metabolite structure prediction which is based on SoMs predicted with SMARTCyp. A further example of a free model is the MetaTox web server (Rudik et al., 2017) which uses a Bayesian approach for the prediction of likely metabolites and additionally estimates the toxicity of predicted metabolites using QSAR models. MetaPrint2D‐React is an extension to the SoM predictor MetaPrint2D (Adams, 2010) which predicts the structures of likely metabolites based on the occurrence ratio of biotransformations in the BIOVIA Metabolite database. Unfortunately, the MetaPrint2D‐React web service is no longer maintained.

Commercial tools for specificity and SoM prediction usually have functionality to generate metabolite structures from their regioselectivity predictions, including StarDrop, ADMET Predictor, and Percepta. Also the previously discussed pseudo‐docking method MetaSite includes an array of methods for the prediction and assessment of metabolite structures. A leading expert system for the prediction of likely metabolites and structures is Meteor Nexus (Marchant, Briggs, & Long, 2008), which mitigates the combinatorial explosion problem with the 2017 addition of a k‐nearest neighbor approach to prioritize transformations toward those most commonly observed (Marchant, Rosser, & Vessey, 2017). Meteor also includes models for SoM prediction via a reimplementation of SMARTCyp and tools for linking mass spectrometry data with predicted metabolites of phase I and phase II biotransformations.

## 5 CHALLENGES AND OUTLOOK

This review has demonstrated the number and diversity of different approaches to predict xenobiotic metabolism highlighting the fundamental importance of this topic to drug discovery. Machine learning methods are by far the most commonly adopted method to make predictions as they can be usefully applied to large, complex data at an early stage in a drug discovery project. It is hoped that this review will be useful in focusing readers’ attention to those methods that can be readily applied in drug discovery programmes.

The users of metabolism prediction tools are primarily industry‐based but due to confidentiality issues data can often not be submitted to web services across the internet. For the available tools to be useful to industry, they need to be distributed as packages that can be executed within the company's infrastructure. However half of all tools developed in academia are unfortunately only offered as web services. This should be reconsidered to make software developed by academia more useable by industry.

The predictive performance of these methods is strong and is probably approaching the limit based on the quality of the available data. However, annotation (SoM assignment in particular) is time‐consuming and requires expert knowledge hindering the availability of data in the public domain. The amount of data is growing slowly and would be greatly aided by the release of metabolic data by pharmaceutical companies but suffered a significant setback when BIOVIA decided to withdraw the Metabolite database from their product range which formed the basis of many applications.

Similarly, strong classification performance is often reported by new methods but it would be desirable if metabolism prediction tools gave some assessment of their domain of applicability or an indication of the confidence domain of predictions. This functionality is sometimes observed in commercial offerings such as the metabolic lability labels in StarDrop and the SoM propensity scores in ADMET Predictor but is often neglected in academic tools. Furthermore, the comparison of methods is often challenging because many studies do not disclose their training sets and the creation of benchmark datasets would make direct comparison much easier. When presenting performance, it would also be beneficial to routinely make comparisons to pure chance to fairly reflect the predictions being made.

We expect the momentum in metabolism prediction software to be maintained in the foreseeable future due to the importance of this topic to drug discovery but progress will inevitably be hindered by the lack of high‐quality data. Currently, the focus has been on CYP metabolism due to its relevance to phase I drug metabolism but we expect that more attention will turn to enzymes involved in other clearance pathways such as glutathione transferases and sulfotransferases.

## AUTHOR INFORMATION

Jonathan Tyzack was awarded a Ph.D. from The University of Cambridge for his studies into the computational prediction of xenobiotic metabolism by cytochrome P450 enzymes. He is currently a research fellow in structural bioinformatics at the European Bioinformatics Institute, part of the European Molecular Biology Laboratory, studying enzyme structure, function and evolution.

Johannes Kirchmair is an associate professor in bioinformatics at the Department of Chemistry and the Computational Biology Unit (CBU) of the University of Bergen. He is also a group leader at the Centre for Bioinformatics (ZBH) of the University of Hamburg. His research activities focus on the development and application of in silico methods for the prediction of biological activities, metabolic fate and toxicity of xenobiotics.
